# Does academic achievement matter? Linking learning engagement to employment quality among vocational students

**DOI:** 10.3389/fpsyg.2026.1777025

**Published:** 2026-04-21

**Authors:** Yue Chen, Meirong Yue, Yan Liu, Shuai Ma

**Affiliations:** 1 College of Education, Hunan Agricultural University, Changsha, China; 2 College of Primary Education, Hunan First Normal University, Changsha, China; 3 Department of Agricultural Leadership, Education and Communications, Texas A&M University, College Station, TX, United States

**Keywords:** academic achievement, behavioral engagement, cognitive engagement, emotional engagement, employment quality, learning engagement, vocational school

## Abstract

**Introduction:**

This study aims to explore the relationships among learning engagement, academic achievement, and employment quality for vocational school students in China. Learning engagement, defined by cognitive, emotional, and behavioral dimensions, plays a crucial role in academic achievement and employment quality.

**Method:**

Data from 624 graduates of vocational schools in Hunan Province in China were analyzed using structural equation modeling (SEM).

**Results:**

Findings revealed that cognitive, emotional, and behavioral engagement significantly predicted academic achievement, while only cognitive engagement had a direct positive impact on employment quality. Notably, academic achievement was found to have no significant direct effect on employment quality. Urban students reported higher employment quality than their rural peers, emphasizing disparities in educational and economic opportunities. Many graduates lacked formal labor contracts and alignment between their jobs and training, reflecting structural barriers to quality employment.

**Discussion:**

Recommendations include fostering learning engagement through targeted teaching strategies, career preparation education, and policies to reduce urban–rural disparities. Enhancing vocational education quality and promoting access to higher education can improve outcomes for vocational school students. The findings highlight the need for systemic reforms in vocational education to bridge gaps in learning engagement, academic achievement, and employment quality.

## 1 Introduction

Employment is the fundamental cornerstone of a stable livelihood. For students and their parents, the primary goal of investing in education is to achieve higher returns, such as increased income and improved social status, both of which are closely linked to the quality of employment. Identifying the influencing factors of the employment quality among vocational school students is vital to improve the quality of enrollment sources in secondary vocational schools. Some research indicates that macro factors such as national welfare system (O’reilly, 2005), production regimes (Gallie, 2007), labor market system (Fields, 2009; Wial, 1991; Holman, 2013), development of science and technology (Vivarelli, 2014; Frey and Osborne, 2017) and economic globalization (Reinecke, 2006; Nayyar, 2015) have a significant impact on employment quality. Meanwhile, other studies have confirmed that micro factors significantly influence employment quality. Vocational skill is one of the key factors affecting individual employment opportunities and wages (Acemoglu and Autor, 2011; Piasna et al., 2013; Stierm, 2015). Students’ academic achievements and active participation in high school programs are identified as positive factors influencing employment quality. This influence becomes more evident through the mediating effect of perceived satisfaction with high school programs (Roh, 2013). Graduates’ employment quality is an important dimension that affects employment status and job quality (González-Romá et al., 2018). Adolescent school engagement had a persistent, positive impact on adult educational and employment outcomes after individual differences were controlled for. The results are interpreted from the perspective that school engagement serves as a conduit for resources essential to future educational and occupational success (Symonds et al., 2023).

In fact, due to the path dependence of changes in national welfare systems and the long-term nature of economic, scientific, and technological development in China, understanding how personal factors influence employment quality is particularly important, as it may provide more actionable insights for secondary vocational education. However, existing theories emphasize the positive role of learning engagement and academic achievement in improving employment quality predominantly focused on general high education or higher academic education, with significantly less attention paid to vocational education, particularly secondary vocational education. With the transformation of industrial structure and the upgrading of vocational education, the labor market’s demand for vocational graduates has gradually shifted from focusing on cognitive skills and academic performance to valuing comprehensive quality, practical operational capabilities, and non-cognitive skills (e.g., communication, teamwork, and adaptability). This shift has weakened the direct correlation between learning engagement, academic achievement and employment quality to a certain extent. Based on the above, the purpose of this study is to examine the current state of employment quality among Chinese vocational secondary school graduates, explore whether and how learning engagement affects their employment quality, and propose recommendations for improving employment quality by enhancing students’ learning engagement and academic achievement. The following research question guided this study:

*RQ*: What is the relationship among learning engagement (cognitive engagement, emotional engagement, behavioral engagement), academic achievement and employment quality?

## 2 Literature review

### 2.1 Employment quality

The concept of employment quality can be traced back to “Decent Work” proposed by the International Labor Organization (ILO), which means “to promote opportunities for women and men to obtain decent work and productive work in conditions of freedom, equity, security and dignity” (ILO, 1999). In 2007, the Conference of European Statisticians made a specialized working group including Eurostat, Eurofound, ILO and UNECE, to measure the quality of work in nine countries including Germany, Canada, France, Finland, Hungary, etc. They released the “Measuring Quality of Employment: Country Pilot Report”, which includes seven dimensions “safety and ethics of employment, income and benefits from employment, working time and work-life balance, security of employment and social protection, social dialogue, skills development and training, employment-related relationships and work motivation (UNECE, 2010). In 2015, On the basis of the aforementioned research, UNECE published the “Handbook on Measuring Quality of Employment: A Statistical Framework”, retaining the 7 dimensions but changing 12 secondary indicators (UNECE, 2010). In 2017, On the basis of recognizing that income and social security are important dimensions of employment quality, OECD emphasizes the working environment, which includes Physical and social environment, Job tasks, organizational characteristics, Working-time arrangements, Job prospects, Intrinsic job aspects (OECD, 2017). In 2017, The Employment Quality Assessment Report released by the Eurofound includes seven indexes: physical environment, work intensity, working time quality, social environment, skills and discretion, prospects and earning (Eurofound, 2017). From all above, it is clear that the employment quality evaluation index system increasingly focuses on the material needs and psychological well-beings of individual workers. Therefore, The International Conference on Labor Statistics (ICLS) points out that employment quality can be interpreted from three levels: social, corporate, and individual. On the individual level, high-quality employment is defined in terms of high wages, job security, guaranteed tenure, and the fulfillment of needs (ICLS, 2018).

In the scholarly literature, quality of employment is a comprehensive concept that includes both macro and micro levels. At the macro level, high-quality employment is characterized by three key elements: sufficient employment opportunities, a rational employment structure, and a robust labor security system (ICLS, 2018). At the micro level, it encompasses various dimensions: wages, standard forms of employment, working time and work-life balance, working conditions and job security, access to training and career development, and collective interest representation (Leschke and Watt, 2014). The employment quality at the micro level can be further defined in both objective and subjective ways (Spencer, 2013). The objective definition refers to the targeting specific objective aspects of work quality—e.g. pay, skill and autonomy, while the subjective definition refers to the well-being at work which relies on the self-reported job satisfaction of employees (Schroeder, 2007; Sinha, 2012; Green et al., 2013; Brendan et al., 2014; Van Aerden et al., 2016; Sehnbruch et al., 2020). It is worth noting that compared to only studying objective or subjective dimensions, comparing and analyzing the weighted composite employment quality index of subjective and objective indicators under a unified framework is more conducive to providing different solutions based on the demands of different labor groups.

From all above, the definition of employment quality has the following three characteristics. One is comprehensiveness, that is, the quality of employment needs to be measured through multidimensional indicators; The second is composite, which means that the quality of employment needs to be measured by both objective indicators and subjective satisfaction from the employees’ self-report; The third is dynamism, which means that the quality of employment is not constant. By improving their technical skills and employment quality, individual workers can achieve a transition from low-quality employment to high-quality employment.

To explore the relationship between learning engagement, academic achievement, and employment quality, the employment quality in this paper considers indexes such as labor contract, workplace, type of the employer, and occupation type at the individual level.

### 2.2 Learning engagement

Learning engagement refers to the attitude of students showing enthusiasm and persistence towards learning. It contains vigor, dedication and absorption, reflecting a high level of energy and a strong sense of identity in learning, as well as a high level of concentration energy (Schaufeli et al., 2002). And it also means the students’ participation in programs and activities that institutions provide for their learning and personal development. It is a multifaceted construct with behavioral, emotional, and cognitive dimensions (Fredricks et al., 2004; Harper, 2008). One of the three dimensions can influence on the other two dimensions. Behavioral engagement can influence and predict emotional and cognitive engagement (Quiment and Smallwood, 2005). Behavioral engagement is a prerequisite for skill development, positive social interaction, and emotional engagement (Martin, 2007).

First, cognitive engagement incorporates motivation, effort, and strategy use (Fredricks et al., 2004), which is related to learning effectiveness. Its essence is that learners use cognitive strategies to regulate their level of self-learning (Wang et al., 2019). Some scholars believe that cognitive engagement refers to the deep psychological and cognitive investment of learners during learning, which refers to the cognitive level of learners after thinking (Ravindran and Debacker, 2005). Cognitive engagement includes students using appropriate learning strategies and investing necessary energy to understand complex problems or master complex skills (Fredricks and McColskey, 2012). Compared to other two dimensions, cognitive investment emphasizes more on the application of subjective willpower, effort, and thinking strategies (Xie et al., 2019).

Second, emotional engagement includes interest, values, and emotions (Fredricks et al., 2004). It can be defined as positive emotional experiencing the value of learning and strong interest in it (Lee, 2008). Moreover, emotional engagement in learning can be divided into two parts: intrinsic emotional engagement and extrinsic emotional engagement. The former refers to the affective states or feelings such as happiness, interest, bore, sadness and zest in the class context (Appleton et al., 2008; Wang et al., 2014; Pöysä et al., 2018; Manwaring et al., 2017), while the latter includes satisfaction with the school, a sense of belonging to the school, and a good relationship with teachers (Kahu, 2013; Wang et al., 2011; Wimpenny and Savin-Baden, 2013; Witkowski and Cornell, 2015).

Third, behavioral engagement encompasses doing the work and following the rules (Fredricks et al., 2004). It includes effort, intensity, persistence, determination, and perseverance in the face of obstacles and difficulties (Skinner and Pitzer, 2012). Behavioral engagement can be seen as the degree of positive interaction between learners and the learning environment, such as teacher-student interaction, peer interaction, group cooperative learning, etc. (Wu and Zhang, 2018). Compared with emotional and cognitive engagement, behavioral engagement relates to the amount of time, persistence, effort, and participation in learning, often relates to the academic achievement and abilities (Miles and Stipek, 2006).

### 2.3 Academic achievement

Academic achievement refers to the knowledge, skills, abilities, or behavioral thinking habits exhibited by learners through learning, which have multiple measurement perspectives such as cognition, emotion, and skill (CHEA, 2003). It requires a planned and organized learning experience, that is, formal school activities. Therefore, academic achievement in a broad sense refers to the knowledge or abilities developed by students in the process of learning with the goal of acquiring knowledge and skills (Adam, 2004). Compared to narrower terms like “academic achievement” and “learning outcome,” this concept has a broader connotation. It typically encompasses academic performance, educational achievement (e.g., dropout or progression rates), and cognitive development (e.g., performance on standardized tests or self-reported gains in knowledge and skills). Therefore, measuring academic achievement should not be limited to the performance of a learner in a particular subject, but rather the comprehensive performance of multiple subjects. Some scholars tried to use regular grades (Hamal, 2021) to measure academic achievement, which is not enough.

The measurement dimensions of academic achievement should be diverse and oriented towards educational goals, which can include knowledge, emotion and skills in a broad sense, as well as exam and test scores in a narrow sense; It can be measured by both teacher observation and evaluation and students’ self-awareness and self-evaluation. Therefore, the evaluation criteria for academic achievement can include subjective and objective indicators. Objective indicators mainly include scores in important unified exams, professional rankings, and the acquisition of vocational skill qualification certificates which are very important for students in vocational education. Subjective indicators are learners’ self-reported gain in knowledge, skills, emotions, attitudes, values, and other aspects after a certain period of learning.

### 2.4 Learning engagement and academic achievement

Learning engagement is an important factor that influences the learners’ academic achievement (Liu et al., 2024). Studies have demonstrated that learning engagement is positively correlated with academic achievement. For instance, Afzal and Crawford (2022) found that student engagement is closely related to their academic achievement in online learning (Afzal and Crawford, 2022). And Andrew et al. showed that there is a significant positive correlation between learning engagement and learning satisfaction, and learning satisfaction is one of the important factors affecting academic achievement through investigation (Andrew et al., 2015). Another study declared that there were positive correlations between student engagement (cognitive engagement, behavioral engagement, and emotional engagement) and English achievement in the domain of college English education for non-English majors in China (Mengjie et al., 2023). Moreover, different types of engagement have been associated with academic achievement. For instance, studies have shown that emotional engagement correlated to educational outcomes, or positively related with academic achievement and learning satisfaction (Connell et al., 1994; Halverson and Graham, 2019; Fredricks et al., 2004), there are a few studies on the emotional engagement’s impact on learning achievement, and some of them find that emotional engagement plays a crucial role in predicting academic achievement (Raver, 2002; Bierman et al., 2008; Liew et al., 2008; Engels et al., 2021; Hasnine et al., 2023). Emotional engagement influences academic achievement through behavioral shaping as a mediator (Geertshuis, 2019) or effect on learning achievement by the interaction with cognitive engagement (Liu et al., 2022). In addition, behavioral engagement can predict academic achievement in E-learning (Kokoç, 2019). And the research revealed that academic achievement is closely linked to the level of student engagement (time spent on homework, time management, and amount of teacher-assigned homework done) of Spain Compulsory Secondary Education (CSE) students (Suárez et al., 2019). Besides, learning goal scores have been found positively correlated with meaningful cognitive engagement (self-regulation and deep strategy use) (Greene and Miller, 1996). And according to Galikyan and Admiraal (2019), there was a positive correlation between cognitive engagement and academic achievement.

### 2.5 Learning engagement and employment quality

The impact of learning engagement on employment quality is an important theme in educational research and career development. According to Dacre Pool and Sewell (2007) learning commitment is one of the important factors in cultivating graduates’ employment quality. Research have shown that there is a significant relationship between how engaged students are in the learning process and their future employment quality (Schunk and Zimmerman, 2007).

### 2.6 Learning achievement and employment quality

Academic achievement can be seen as an indicator of the knowledge and skills students acquire in the education system, and these factors often affect their employment quality and career progression. Studies have suggested a positive correlation between learning achievement and employment quality. For instance, Dacre Pool and Sewell (2007) put forward the employment quality model of graduates, emphasizing the relationship between academic achievement and employment quality, pointing out that high academic achievement helps to improve employment opportunities and the possibility of career development. It has been certified that there is the significant relationship between academic achievement and self-concept with employment quality (Muhamad, 2019). And some research indicate that academic achievement is an important factor in assessing the quality of employment, and also conform the obvious relationship between academic achievement and employment quality (Hillage and Pollard, 1998; Woods and Datchi, 2018). According to Choi (2018) academic achievement has been found to positively affect job satisfaction. Furthermore, it certified that there is the positive relation between students’ academic achievement in school and earnings in the future labor market. For example, according to the Cognitive Skills (Human Capital) Theory, if a college education provides cognitive skills that lead to productivity in the workplace, then a positive relationship should exist between academic achievement and earnings (Gary, 2004). And the research analyzed how students’ academic achievement impacts future labor markets rewards, and found that there is a significant positive impact of GPA on salaries of BA graduates (9–12% wage premium for an additional GPA point) (Victor and Sergey, 2019). However, some research in China indicated that academic performance has no significant impact on graduates’ starting salary, employment satisfaction, or the alignment (Bao, 2022). The data from the 2015 National Survey on the Employment Quality of Secondary Vocational Education Graduates in China examined the impact of non-cognitive skills on graduates’ wages and employment stability. The findings reveal that the return rate for non-cognitive skills on wages is nearly double that of cognitive skills, leading to the conclusion that non-cognitive skills have a significant positive impact on employment quality (Li et al., 2019).

## 3 Hypothesizes and proposed model

**Figure 1:**
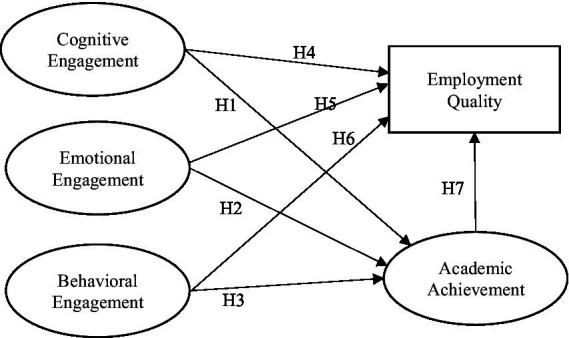
Proposed model.

The study aims to investigate the relationship among learning engagement (cognitive engagement, emotional engagement and behavioral engagement), academic achievement, and employment quality. Figure 1 demonstrated the proposed model. The proposed hypothesizes in the model are as follows:

*H1*: There is a direct positive relationship between cognitive engagement and academic achievement.

*H2*: There is a direct positive relationship between emotional engagement and academic achievement.

*H3*: There is a direct positive relationship between behavioral engagement and academic achievement.

*H4*: There is a direct positive relationship between cognitive engagement and employment quality.

*H5*: There is a direct positive relationship between emotional engagement and employment quality.

*H6*: There is a direct positive relationship between behavioral engagement and employment quality.

*H7*: There is a direct positive relationship between academic achievement and employment quality.

## 4 Methods

### 4.1 Participants

The data comes from the 2024 “Employment Quality Survey of Vocational School Students” conducted by [Hunan Agricultural University]. [Hunan Agricultural University] is located in the central region of China. The Education College was approved by the Ministry of Education in 1999 as a “National Key Vocational Education Teacher Training Base”. It has hold 28 undergraduate vocational teacher education majors, which work as teachers in various secondary vocational schools in Hunan Province now. Considering the significant differences in employment quality in Chinese different regions, and to avoid the impact of different employment years, this survey adopts purposive sampling and stratified random sampling methods, that is, we randomly choose some of the vocational school teachers who graduated from [Hunan Agricultural University] and asked them to distributes questionnaires to their own students who graduated in 2020, 2021, 2022, and 2023 through the Chinese online questionnaire platform called Wenjuanxing. The participants have been employed for 1–4 years in Hunan Province from different majors. Before collecting the data, ethical approval was obtained from the ethics committee in [Hunan Agricultural University]. The students signed informed consent before participating. Finally, 708 questionnaires were collected, after removing missing data, 624 valid questionnaires were analyzed. The survey was distributed in Chinese and the English version of the survey was provided in Appendix. A comprehensive overview of the subjects’ demographic information is presented in Table 1.

**Table 1:** Demographic information of participants (*n* = 624).

Variables	Group	*N*	Frequency (%)
Gender	Male	349	55.93
	Female	275	44.07
Household registration	Urban	52	8.33
	Rural	572	91.67
Father’s education	Illiterate	24	3.85
	Primary school	87	13.94
	Junior high school	381	61.06
	Secondary school	114	18.27
	Undergraduate	15	2.40
	Postgraduate	3	0.48
Mother’s education	Illiterate	40	6.41
	Primary school	118	18.91
	Junior high school	357	57.21
	Secondary school	96	15.38
	Undergraduate	13	2.08
	Postgraduate	0	0.00
Family’s income	Poor	31	4.97
	Relatively poor	109	17.47
	Middle	469	75.16
	Relatively rich	11	1.76
	Rich	4	0.64

### 4.2 Measures

We used survey to collect learning engagement, self-reported academic achievement, and employment quality. The survey also contains demographic information like gender, family registration type and family income.

Employment Quality Scale (see Appendix 1). Due to differences in traditional culture and socio-economic structures, the employment quality views in China and the West are not equivalent. So, we searched 67 papers from CSSCI (Chinese Social Sciences Citation Index), 30 papers in Web of Science and 8 report from international organizations under the title of “quality of employment”, “quality of job”, “decent work” or “quality of working”. We coded all the indicators from these 97 papers and reports based on the Grounded Theory. There are 14 indicators which are mentioned most frequently. We tried to translate them into Chinese context, then they are 4 objective questions about labor contract, nature of employer, social security and occupation type. We employed those four indicators and calculated a number for employment quality index based on the mainstream research methods in academia (Leschke and Watt, 2014). We constructed an Employment Quality Index based on four indicators: labor contract, workplace, type of the employer, and occupation type. First, we performed range standardization on the four indicators to obtain the standardized shared indicators. The formula used is:


$$x_{\mathrm{ij}}^{\mathrm{equ}}=\frac{x_{\mathrm{ij}}-\min_{j}\;}{\mathrm{ma}x_{j}-\mathrm{mi}n_{j}}$$

where *i* represents the individual subject, *j* represents a specific measurement indicator, and *min_j_* and *max_j_* are the minimum and maximum values of the indicator, respectively. On this basis, we used the equal weight averaging method to calculate the Employment Quality Index. The formula is as follows:


$$\text{qualit}y_{i}=\frac{1}{4}\sum_{j=1}^{4}x_{\mathrm{ij}}^{\mathrm{equ}}.100$$

Academic Achievement Scale (see Appendix 2). From the subjective perspective, students’ self-report of learning outcomes is an important indicator of academic achievement, which can be divided into multiple dimensions such as cognition, skills, emotion, attitudes, and values. Finally, we designed a Chinese secondary vocational school student academic achievement scale consisting of self-reported subjective items (Likert scale).

Learning Engagement Scale (see Appendix 3). Studies have shown that learning engagement can affect academic achievement and employment quality. Researching three dimensions of learning engagement, referring to “School Engagement Scale” (Maroco et al., 2016), a questionnaire was designed, which includes three parts: cognitive engagement, emotional engagement, and behavioral engagement (ECC27 about homework is deleted because Chinese secondary vocational school students in “class for job” not “class for admission” have no homework in usual), with a total of 31 questions. Respondents were asked to self-report their learning engagement during their vocational school years with 5-point Likert scale. (1 = Completely disagree; 2 = Not quite agree; 3 = Uncertain; 4 = Comparatively agree; 5 = Completely agree).

Demographic information was also embedded in the survey. Gender, Family Income, Household Registration Type. There are studies showing that employment quality is influenced by gender, family income, household registration type (urban or rural) (Zhang et al., 2021). Therefore, gender, family income, urban or rural household registration, are used as control variables to reduce the influence of omitted variables on the research results. (1) Gender: Assign “1” to males and “0” to females. (2) Household registration type: Assign “urban” as “1” and “rural” as “0.” (5) Family Income. (1 = poor, 2 = relatively poor; 3 = middle/not poor nor rich; 4 = relatively rich; 5 = rich). 1, 2 was further recoded as low income and 3, 4, 5 were further recoded as non-low-income status.

### 4.3 Data analysis methods

Descriptive analysis was run in STATA (18.0). Mplus software (VERSION 8.1) was employed for exploratory factor analysis (EFA) and Confirmatory factor analysis (CFA), Structural Equation Modeling (SEM) to investigate the model and relationship among factors.

## 5 Results

### 5.1 Descriptive result

The employment quality of Chinese vocational school students is not high based on the date we collected. We measured employment quality scale through labor contract, nature of the employer, social security and occupation type. However, From the questionnaires we collected, it appears that 69.53% of the vocational school students did not sign a labor contract, they do not know much about China’s social security system or what they can get from it, none of them work in government or government-affiliated institutions, 69.06% of them do not know their nature of the employer. 45.16, 16.25, 28.91, 9.84% of them are, respectively, non-skilled workers, semi-skilled workers, skilled workers and self-employed worker, none of them are officer or manager.

### 5.2 Measurement model

We include cognitive engagement, emotional engagement, behavioral engagement and academic achievement as four latent variables in the measurement model. Initially the 39 items reached a reliability of a = 0.9787. The factor loadings range from 0.45 to 0.86 and loaded on four distinct factors. For the measurement model, we used the following criteria for fit indices, including the root mean square error of approximation (RMSEA) with a threshold value of 0.08 (Hu and Bentler, 1999) standardized root mean squared residuals (SRMR) with a threshold value of 0.08 (Byrne, 1994), and the comparative fit index (CFI) with a threshold value of 0.90. Initially the index suggested an inadequate fit (RMSEA = 0.081, CFI = 0.889, SRMR = 0.043). Based on the factor loading indices, we investigated the items with low factor loading and cross loading, we deleted Item 8 from academic achievement, Item 4, Item 5, Item 6 from cognitive engagement scale, as well as Item 1 from behavioral engagement scale. The removal of the five items did not materially narrow the conceptual breadth of the affected scales. In each case, the deleted items were either peripheral to the core construct or conceptually overlapping with remaining items. The remaining items continue to comprehensively represent the theoretical domains of academic achievement, cognitive engagement, and behavioral engagement; thus, the overall construct coverage of the scales remains intact. After that we allow correlation between error terms of specific items, namely the error terms of three pairs correlate with each other (Item 2 and Item 3 from emotion scale, item 4 and 5 from emotion scale, item 8 and 9 from emotion scale), After the model re-specification, we reached an acceptant fit for measurement model (χ^2^ (518) = 2445.21, *p* < 0.001, RMSEA = 0.077, CFI = 0.901, SRMR = 0.041). Table 2 demonstrated the descriptive statistics of the modified instruments. The modified items reached the reliability of a = 0.9754 with 34 remaining items.

**Table 2:** Descriptive statistics (*n* = 624).

Variable	Cognitive engagement	Emotional engagement	Behavioral engagement	Academic achievement	Employment quality
Mean	23.31	36.48	36.97	25.74	25.63
SD	5.34	9.62	9.33	6.49	20.88
Min	6	10	10	7	0
Max	30	50	50	35	75
Cronbach’s alpha	0.9251	0.9478	0.9512	0.9186	

*p < 0.05, **p < 0.01, ***p < 0.001.

### 5.3 Structural model

A Structural Equation Modeling (SEM) was constructed with cognitive engagement, emotional engagement and behavioral engagement as the independent variable, and academic achievement and employment quality as dependent variables. Gender, urban status and family income were treated as control variables in the model where those control variables have impact on academic achievement and employment quality. Meanwhile, the model with academic achievement as predictor and employment quality as outcomes was evaluated. Cognitive engagement, emotion engagement, behavior engagement and academic achievement were designed as latent variables. In the structural model, the model demonstrated a good fit (χ^2^ (647) = 2597.55, *p* < 0.001, RMSEA = 0.070, CFI = 0.900, SRMR = 0.040).

**Figure 2:**
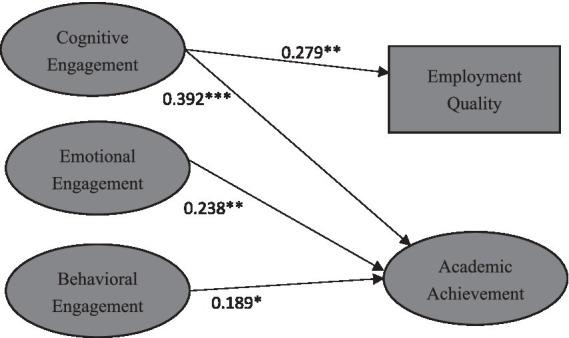
Model result. *p < 0.05, **p < 0.01, ***p < 0.001.

Table 3 demonstrated the results of the structural model. Notably, we discovered significant positive relationship between cognitive engagement and academic achievement (*β* = 0.392, *p* < 0.001), emotional engagement and academic achievement (*β* = 0.238, *p* < 0.01), behavioral engagement and academic achievement (*β* = 0.189, *p* < 0.05) indicating all three types of engagement are significant predictors of academic achievement. As for the relationship between engagement and employment quality, we only found significant positive relationship between cognitive engagement and employment quality (*β* = 0.279, *p* < 0.01) indicating cognitive engagement being a significant predictor on employment quality. On the contrary, emotional engagement (*β* = −0.225, *p* = 0.056) and behavioral engagement (*β* = 0.099, *p* = 0.376) was not significant. Notably, we did not find a significant relationship between academic performance and employment quality (*β* = −0.109, *p* = 0.113). H1, H2, H3, H4 were supported but H5, H6, H7 were not supported. Figure 2 demonstrated the model results with significant relationships.

**Table 3:** Hypothesis results.

Hypothesis	Direct effect	*β*	*p*
H1	Cognitive engagement → academic achievement	0.392	0.000***
H2	Emotional engagement → academic achievement	0.238	0.004**
H3	Behavioral engagement → academic achievement	0.189	0.018*
H4	Cognitive engagement → employment quality	0.279	0.001**
H5	Emotional engagement → employment quality	−0.225	0.056
H6	Behavioral engagement → employment quality	0.099	0.376
H7	Academic achievement → employment quality	−0.109	0.113

*p < 0.05, **p < 0.01, ***p < 0.001.

As for the control variables, we only found significant relationship between Urban status and employment quality (*β* = 0.110, *p* = 0.005). We did not find significant relationship between gender and employment quality (*β* = 0.011, *p* = 0.779) nor the relationship between family income (*β* = −0.060, *p* = 0.126) and employment quality. No significant relationship was found between gender on academic achievement (*β* = 0.012, *p* = 0.669), urban status on academic achievement (*β* = −0.007, *p* = 0.812), and family income on academic achievement (*β* = −0.021, *p* = 0.466).

## 6 Discussion and recommendations

This study investigates the relationship among learners’ learning engagement (cognitive engagement, emotion engagement, behavior engagement), academic achievement and employment quality in secondary vocational schools. It revealed the following findings: (1) There are significant differences in the employment quality of vocational school students based on their household registration types, students with urban household registration have higher employment quality compared to their rural peers. De Brauw et al. (2002) show that compared with the international average rate of return to education of 10.1%, the rate for rural students in China is generally between 0 and 6%. Some Chinese scholars have also confirmed that there is a significant gap in the education return between urban and rural areas in China based on data such as the China Household Income Survey (CHIP). This may be due to the gap of economy and education quality between urban and rural areas in China, as well as the gap of social capital between urban and rural families. (2) The learning engagement of vocational school students can positively predict academic achievement, cognitive engagement, emotional engagement, and behavioral engagement are all positively correlated with the academic achievement of vocational school students. This is consistent with the relationship between learning engagement and academic achievement in other types and stages of education. Knowledge is externalized to students, and only when students make psychological investment and effort in mastering, understanding, and acquiring skills, can knowledge be known, mastered, and applied by students. Only when vocational school students realize the importance of vocational education, use learning strategies, maintain a positive emotional state, and are willing to take action for it, can they make good academic achievements. (3) There is a positive correlation between cognitive investment and employment quality among vocational school students. The positive link between cognitive engagement and work success is supported by the direct path from cognitive engagement to employment quality in our structural model. This conclusion is supported by the positive link between cognitive engagement and work adaptation, as documented in Li et al. (2023) and Daan et al. (2025) in the context of Chinese vocational training for vulnerable groups. Cognitive engagement emphasizes more on the application of subjective willpower, effort and thinking strategies. This subjective willpower and effort not only occur in learning but also in the early career stage. It not only affects learning achievement, but also helps learners to continue striving in the work.

From all the above findings, and considering the low-quality of vocational school students’ employment, we propose the following recommendations for the students, teachers and administrator: (1) Improve the quality of secondary vocational education and strengthen cognitive engagement, rather than simply pushing students to pursue higher education. Administrators should develop relevant policies to improve teaching quality and learning engagement in secondary vocational schools. For example, continue to implement “secondary vocational education free” policies, reduce personal costs of vocational education, open up a specialized admission channel for the vocational students, improve the quality of education so that students can achieve high employment quality without necessarily upgrading to college education. (2) Improve the employment quality of rural students, narrow the gap of secondary vocational education return between urban and rural areas. First, we should continue to take “narrowing the gap between urban and rural areas” as the ruling direction and political goal of the government. Second, we should increase investment in rural education, improve its quality, so that the students can obtain technology and improve their employment quality through education. Finally, we should rely on the rural revitalization policy, implement employment assistance for rural students, eliminate household discrimination and urban and rural mobility system barriers, and the gap in urban and rural employment quality caused by the registered residence system. (3) Strengthen the cognitive, emotional, and behavioral engagement of secondary vocational school students, and improve their academic achievement and employment quality. Firstly, the government and schools should rev up publicity on the importance of secondary vocational education, informing students of the importance of secondary vocational education in improving their skills and employment quality. Students use cognitive strategies to focus on learning, prepare well before class, actively participate in group activities, maintain harmonious relationships with teachers and classmates, and help each other. Teachers should enhance their teaching abilities, utilize modern teaching techniques, make teaching activities more engaging, maintain a positive emotional state both inside and outside the classroom, and strengthen students’ sense of belonging to the school and class. Finally, the school offers relevant lectures on learning methods. When students have difficulties in learning, teachers should help them reflect, encourage them to continue working hard, and help them understand and apply knowledge in practice. (4) Enhance the cognitive engagement of secondary vocational school students and improve their employment quality. Firstly, individual learners should establish correct learning ideas and attitudes, abandon the utilitarian mentality of “vocational education is useless”, establish an internal pursuit of expanding knowledge and improving abilities through learning to achieve self-worth, consciously cultivate self-learning and self-management abilities, and actively adjust their psychological state and learning strategies. Secondly, teachers should adopt the “Twenty-One Teaching Strategies” (Kimberly, 2013), teach for all students, give students opportunities to think and talk, and encourage, demand, and actively manage the participation of all students, build an inclusive and fair classroom community, monitor behavior to cultivate divergent thinking. Finally, the school carries out career planning education, guiding students to develop learning plans, identify employment goals and life directions, and have a correct understanding and grasp of their studies, life, work, etc. guiding them to the right track, helping them correct their mistakes, avoiding them from getting lost, and maximizing their gains in school.

While the finding that a high percentage of vocational school graduates lacked formal labor contracts highlights a serious vulnerability in their early labor-market experiences. Many of the challenges reflect broader structural features of China’s labor and social welfare systems and the need for coordinated action from employers, regulatory agencies, and the wider social security framework, however, vocational schools can still play an important role in strengthening career readiness education, including basic labor law literacy, contract awareness, and social insurance knowledge. Such efforts can improve students’ awareness and ability to navigate the labor market.

We did not find a significant relationship between academic achievement and employment quality. In the field of vocational education in China, factors influencing employment quality also encompass institutional characteristics, family socioeconomic status, and internship experiences (Bao, 2022). Non-cognitive skills of secondary vocational graduates have a significant positive effect on wages, an effect larger than that of cognitive skills, and that non-cognitive skills can significantly enhance employment stability (Li et al., 2019). Future research should continue to investigate the relationship between academic achievement and employment quality and the mediator factors/variables, or in higher education.

## 7 Limitation

### 7.1 Questionnaire quantity and the scope of survey

Due to the different economic development levels and different labor markets, there are big disparity in earning among different provinces in China. As well as significant differences in employment quality among vocational school students in different years caused by diploma devaluation. Therefore, the research subjects had to be limited to vocational school students who graduated in the same province within the past 4 years, and only 624 valid questionnaires were collected. Although it has certain representativeness and reliability, if we can conduct a larger survey and obtain more samples, it will help us to have a clearer understanding of the employment quality of vocational school students in China, as well as the impact of learning engagement and academic achievement on employment quality, and the correlation between various internal factors. Our sample was drawn from a single province in China, so the generalizability of the findings should be interpreted with caution. Future research could replicate this study by including additional provinces or even conducting a nationwide survey to further validate our results. Further study should strive for more funding and social support as much as possible, expand the scope of research, and increase the sample size.

Limitation on the academic achievement measure. We only employed self-reported academic achievement in the model. From the objective perspective, achieving at least one vocational qualification certificate is a very important educational aim for secondary vocational school students. At the same time, considering the difficulty of comparing grades between different majors, professional ranking is considered as an important reference for academic achievement.

### 7.2 Chinese characteristics causes limited applicability of the recommendations

China’s political, economic, and welfare systems have distinct characteristics, the quality of employment of vocational school students were in high-level in the first few decades of Founding of the People’s Republic of China. But, in recent years, the relative economic and non-economic benefits of secondary vocational education have significantly declined because of the expansion of higher education enrollment and diploma devaluation. Secondary vocational education has become a forced choice for children with poor academic achievement from economically disadvantaged rural families before their legal working age. Their low-level learning engagement, poor academic achievement, and low employment quality have formed a vicious cycle. To break this vicious cycle, the Chinese government implements the strategy of rural revitalization, strengthens the quality construction of secondary vocational education, encourages secondary vocational students participate in learning, improves the vocational education college entrance examination system, enhances the quality of higher vocational education, provides diversified promotion channels for secondary vocational graduates. However, these practices require strong support from centralized government and socialist system, so applicability of our recommendations may have limitation of generalization for other countries and regions. Although the recommendations offered in the discussion section are practical within the Chinese context, they rely heavily on state-led governance structures and policy mechanisms that may not exist in other countries. As a result, their applicability to international settings should be interpreted cautiously, as different institutional and regulatory environments may limit direct transferability.

## Data Availability

The original contributions presented in the study are included in the article/Supplementary material, further inquiries can be directed to the corresponding author/s.
