# Supplementary material for: Does academic achievement matter? Linking learning engagement to employment quality among vocational students
Source: Front Psychol. 2026 Apr 21;17:1777025. doi: 10.3389/fpsyg.2026.1777025 (PMC13139066; doi:10.3389/fpsyg.2026.1777025)
Supplement: Supplementary file 1 [file Supplementary_File_1.docx]

**Appendixes**

Appendix 1 Chinese Secondary Vocational School students' Employment Quality Scale

| **QUESTIONS** | **SCORE** | | | | | | | |
| --- | --- | --- | --- | --- | --- | --- | --- | --- |
| 1.Did you sign a labor contract for your job? | 1=Signed | | | | 0=Didn’t Sign | | | |
| 2.What’s the nature of your employer? | 1=unknown | 2=private-owned enterprise | | 3= state- owned enterprise | | 4=government-affiliated institutions | | 5=government |
| 3.How much your employer paid for your five insurances and one fund? | 1=No/never heard | 2=a little of them | | 3=some of them | | 4=most of them | | 5=all of them |
| 4.What’s your occupation type? | 1=Non-skilled Worker | 2=Semi-skilled Worker | 3= Skilled Worker | | 4=Self-employed Worker | | 5=Officer | 6=Manager |

Note: The variables ‘employer nature’ and ‘occupational type’ were coded on ordered categorical scales that reflect the relative status and stability of employment within the Chinese labor market. For employer nature, higher scores indicate more formal and prestigious sectors, with government and government‑affiliated institutions traditionally regarded as offering the strongest job security and welfare benefits. Similarly, the occupational type scale progresses from non‑skilled to managerial roles, representing increasing levels of skill, responsibility, and labor‑market advantage. These ordered categories were treated as continuous indices to capture the underlying gradient of employment quality and occupational status in a way that aligns with Chinese institutional and cultural norms.

Appendix 2 Chinese Secondary Vocational School students' Academic Achievement Scale

QUESTIONS (1=Completely disagree; 2=Not quite agree; 3=Uncertain; 4=Comparatively agree; 5=Completely agree)

1. The study in secondary vocational school allowed me to acquire rich knowledge and develop my thinking ability.

2. I have mastered the vocational skills related to my major during the secondary vocational education.

3. I have mastered effective learning methods and formed good learning habits during the secondary vocational education.

4. I met a lot of people and developed relationships during the secondary vocational education.

5. I have improved my independent learning ability during the secondary vocational education.

6. I developed an interest in learning during the secondary vocational education.

7. I have formed a correct world outlook, outlook on life and values during the secondary vocational education.

8. I have strengthened the consciousness of patriotism and law-abiding, and improved the moral cultivation during the secondary vocational education. (deleted)

Appendix 3 Chinese Secondary Vocational School Students' Learning Engagement Scale

(1-10 = "cognitive engagement"; 11-20 = "emotional engagement"; 21-31= "behavioral engagement"; score 1=Completely disagree; score 2=Not quite agree; score 3=Uncertain; score 4=Comparatively agree; score 5=Completely agree)

Cognitive engagement (10 items)

1. I concentrated on the class during the secondary vocational education.

2. I tried to complete the study task in secondary vocational class.

3. I obeyed the rules of the class during the secondary vocational education.

4. I asked questions in class and participate in debates when I have doubts during the secondary vocational education. (deleted)

5. I usually took an active part in group work during the secondary vocational education. (deleted)

6. I usually learned the material recommended by the teacher before going to class during the secondary vocational education. (deleted)

7. I got along well with the teachers during the secondary vocational education.

8. I got along well with other students during the secondary vocational education.

9. I asked my classmates for help when I did not understand the teaching content during the secondary vocational education.

10. I helped my classmates when they ask me my questions during the secondary vocational education.

Emotional Engagement (10 items)

1. I took part in extra-curricular activities at school (concerts, exhibitions, lectures, conferences, etc.) during the secondary vocational education.

2. I was very happy in class during the secondary vocational education.

3. I felt that learning the course of the school is very fulfilling during the secondary vocational education.

4. I was excited about learning the school curriculum during the secondary vocational education.

5. I liked school lessons during the secondary vocational education.

6. I was interested in learning content during the secondary vocational education.

7. I usually talked to the instructor about my professional interests, development direction or career plans during the secondary vocational education.

8. The school classroom is an interesting place during the secondary vocational education.

9. I participated in extracurricular activities (club activities, lectures, meetings, etc.) with other students after class during the secondary vocational education.

10. I discussed with my classmates how to make the class better during the secondary vocational education.

Behavioral Engagement (11 items)

1. I reflected or questioned myself in class to make sure I understand what is being taught during the secondary vocational education. (deleted)

2. Even if it is not for passing the exam, I studied hard after class during the secondary vocational education.

3. During the secondary vocational education, I tried to watch TV programs about the content of the class, extracurricular books, etc.

4. I discussed what I have learned in class with others outside of class during the secondary vocational education.

5. During the secondary vocational education, If I don't understand a certain knowledge point, I will try to solve the problem, such as looking up literature or asking others.

6. I checked my work to correct mistakes during the secondary vocational education.

7. I tried to combine what I have learned to solve new problems during the secondary vocational education.

8. I read a lot of things related to the class content after class during the secondary vocational education.

9. During the secondary vocational education, If I do not learn, I will go back to learn again.

10. I reviewed after school during the secondary vocational education.

11. I tried to integrate classroom knowledge with other knowledge to integrate my knowledge reserve during the secondary vocational education.

Note: Item 8 from academic achievement, Item 4, Item 5 Item 6 from cognitive engagement scale, and Item 1 from behavioral engagement scale were deleted during the model re-specification process.
